# Supplementary material for: Comparison of Japanese Encephalitis Force of Infection in Pigs, Poultry and Dogs in Cambodian Villages
Source: Pathogens. 2020 Sep 1;9(9):719. doi: 10.3390/pathogens9090719 (PMC7558861; doi:10.3390/pathogens9090719)
Supplement: Supplementary file 1 [file pathogens-09-00719-s001.zip › Table S1.docx]

**Table S1:** Flavivirus and JEV forces of infection (FOI) by species, in Khsach Kandal district only.

|  | **Flavivirus FOI (HIA)** | | **JEV FOI (FRNT)** | |
| --- | --- | --- | --- | --- |
|  | **FOI** | **95% IC** | **FOI** | **95% IC** |
| **Pigs** | 0.12 | (0.09 - 0.16) | 0.12 | (0.09 - 0.16) |
| **Ducks** | 0.06 | (0.04 - 0.09) | 0.05 | (0.04 - 0.08) |
| **Dogs** | 0.02 | (0.01- 0.02) | 0.01 | (0.01 - 0.02) |

**Chickens were excluded because no chicken sample was positive in Khsach Kandal*
