# Supplementary material for: Comparison of Japanese Encephalitis Force of Infection in Pigs, Poultry and Dogs in Cambodian Villages
Source: Pathogens. 2020 Sep 1;9(9):719. doi: 10.3390/pathogens9090719 (PMC7558861; doi:10.3390/pathogens9090719)
Supplement: Supplementary file 1 [file pathogens-09-00719-s001.zip › pathogens-902278-supplementary-english.doc]

**File S1:** Estimation of the flavivirus forces of infection per month in chickens and ducks sampled in Kandal province in 2016.

In order to support the notion that the circulation of JEV in poultry is little affected by seasonality, we could estimate forces of infection (FOI) on the 2016 poultry dataset, which was the subject of Auerswald et al., 2020 [1]. In March 2016, 417 chickens and 203 ducks were blood sampled in three provinces of Cambodia, including Kandal province (n=296). The age was known for 492 (79.4%) domestic birds and ranged from 1 to 36 months (mean 7.93 months; 95% CI 7.19–8.67). All sera were first tested with hemagglutination inhibition assay (HIA) [2] for flavivirus seroprevalence. Only the HIA-positive samples with sufficient serum volume left could be tested by foci reduction neutralization test (FRNT) (n=65) [3]. Overall flavivirus seroprevalence was 23.7% in chickens and 39.9% in ducks. The observed flavivirus seroprevalence differed among the provinces studied and was lowest in Kandal (19.6%, 58/296 samples).

We selected the 296 chickens and ducks that were collected in Kandal province to estimate the FOI on the HIA results of this sample, as our study focused on Kandal province. The FOI estimation methods were the same as the one we used and are detailed in the main paper.

The flavivirus FOI estimates in Kandal were 0.05 per month and 0.03 per month for the 2016 ducks and chickens, respectively (Table 1). As the confidence intervals of estimated Flavivirus FOI do not overlap, the Flavivirus FOI was significantly higher in ducks than in chickens.

**Table 1:** Chickens and ducks flavivirus forces of infection (FOI) per month in Kandal Province in 2016.

|  | **Flavivirus FOI (HIA)** | |
| --- | --- | --- |
|  | **FOI** | **95% CI** |
| **Ducks** | 0.05 | (0.04 - 0.07) |
| **Chickens** | 0.03 | (0.02 - 0.03) |

1.	Auerswald, H.; Ruget, A.S.; Ladreyt, H.; In, S.; Mao, S.; Sorn, S.; Tum, S.; Duong, V.; Dussart, P.; Cappelle, J.; et al. Serological evidence for Japanese encephalitis and West Nile virus infections in domestic birds in Cambodia. *Front. Vet. Sci.* **2020**, *7*, 9.

2.	Clarke, D.H.; Casals, J. Techniques for hemagglutination and hemagglutination-inhibition with arthropod-borne viruses. *Am. J. Trop. Med. Hyg.* **1958**, *7*, 561–573.

3.	Auerswald, H.; de Jesus, A.; Seixas, G.; Nazareth, T.; In, S.; Mao, S.; Duong, V.; Silva, A.C.; Paul, R.; Dussart, P.; et al. First dengue virus seroprevalence study on Madeira Island after the 2012 outbreak indicates unreported dengue circulation. *Parasit. Vectors* **2019**, *12*, doi:10.1186/s13071-019-3357-3.
